# Exploring the Capacity of Large Language Models to Assess the Chronic Pain Experience: Algorithm Development and Validation

**DOI:** 10.2196/65903

**Published:** 2025-03-31

**Authors:** Jacopo Amidei, Rubén Nieto, Andreas Kaltenbrunner, Jose Gregorio Ferreira De Sá, Mayte Serrat, Klara Albajes

**Affiliations:** 1 AI and Data for Society Research Group Internet Interdisciplinary Institute Universitat Oberta de Catalunya Barcelona Spain; 2 eHealth Lab Research Group Faculty of Psychology and Educational Sciences Universitat Oberta de Catalunya Barcelona Spain; 3 Unitat d’Expertesa en Síndromes de Sensibilització Central Servei de Reumatologia Vall d'Hebron Hospital Universitari Barcelona Spain; 4 Escola Universitària de Fisioteràpia Escoles Universitàries Gimbernat Barcelona Spain; 5 Psyclinic Mental Health Barcelona Spain

**Keywords:** large language models, fibromyalgia, chronic pain, written narratives, pain narratives, automated assessment, pain severity, pain disability

## Abstract

**Background:**

Chronic pain, affecting more than 20% of the global population, has an enormous pernicious impact on individuals as well as economic ramifications at both the health and social levels. Accordingly, tools that enhance pain assessment can considerably impact people suffering from pain and society at large. In this context, assessment methods based on individuals’ personal experiences, such as written narratives (WNs), offer relevant insights into understanding pain from a personal perspective. This approach can uncover subjective, intricate, and multifaceted aspects that standardized questionnaires can overlook. However, WNs can be time-consuming for clinicians. Therefore, a tool that uses WNs while reducing the time required for their evaluation could have a significantly beneficial impact on people's pain assessment.

**Objective:**

This study is the first evaluation of the potential of applying large language models (LLMs) to assist clinicians in assessing patients’ pain expressed through WNs.

**Methods:**

We performed an experiment based on 43 WNs made by people with fibromyalgia and qualitatively evaluated in a prior study. Focusing on pain severity and disability, we prompt GPT-4 (with temperature parameter settings 0 or 1) to assign scores and scores’ explanations, to these WNs. Then, we quantitatively compare GPT-4 scores with experts’ scores of the same narratives, using statistical measures such as Pearson correlations, root mean squared error, the weighted version of the Gwet agreement coefficient, and Krippendorff α. Additionally, 2 experts specialized in chronic pain conducted a qualitative analysis of the scores’ explanation to assess their accuracy and potential applicability of GPT’s analysis for future pain narrative evaluations.

**Results:**

Our analysis reveals that GPT-4’s performance in assessing pain narratives yielded promising results. GPT-4 was comparable in terms of agreement with experts (with a weighted percentage agreement higher than 0.95), correlations with standardized measurements (for example in the range of 0.43 and 0.49 between the Revised Fibromyalgia Impact Questionnaire and GTP-4 with temperatures 1), and low error rates (root mean squared error of 1.20 for severity and 1.44 for disability). Moreover, experts generally deemed the ratings provided by GPT-4, as well as the scores’ explanation, to be adequate. However, we observe that GPT has a slight tendency to overestimate pain severity and disability with a lower SD than expert estimates.

**Conclusions:**

These findings underline the potential of LLMs in facilitating the assessment of WNs of people with fibromyalgia, offering a novel approach to understanding and evaluating patient pain experiences. Integrating automated assessments through LLMs presents opportunities for streamlining and enhancing the assessment process, paving the way for improved patient care and tailored interventions in the chronic pain management field.

## Introduction

Chronic pain poses a widespread challenge, affecting more than 20% of the global population [1-4]. Such kinds of pain are associated with restrictions in daily activities, disrupting normal functionality, and diminishing the overall quality of life. Its repercussions extend to disruptions in familial, professional, and social domains [3]. Additionally, persistent pain is often linked to mental health issues, such as depression or anxiety disorders [5]. As reported in several studies [6,7], pain is one of the main reasons for medical consultations, and people with chronic pain use a high quantity of health care services (especially those with disabling pain problems). Accordingly, pain significantly contributes to the demand for medical services, imposing a noteworthy economic burden on both, individuals experiencing the pain and society at large. Therefore, the economic ramifications at both the health and social levels are substantial. For example, estimates suggest that in Spain alone, the annual total cost, encompassing both direct and indirect expenses, could reach €16 billion (US $17.27 billion) [8].

Tools that enhance the assessment to understand better people with pain experiences are essential. Only with an adequate assessment, it is possible to determine the best intervention approach for each person and to evaluate the effects of interventions. Along these lines, standardized instruments (mainly self-reported measures) and established procedures exist that are available and recommended for routine assessment of people with pain. More specifically, experts recommend taking into account different domains (eg, pain severity, interference with daily activities, emotional functioning), and using some specific self-report measurements [9,10]. However, self-reported measures are limited to a specific domain and cannot capture the subjectivity of the pain experience. For this reason, assessment procedures based on people’s own experiences have been claimed as valuable better to understand the pain experience from a personal lens. In fact, a qualitative approach can capture subjective, intricate, and multifaceted details that standardized questionnaires can fail to capture [11]. In the end, pain is always an internal and subjective experience, providing qualitative reports with richer content to understand globally this subjective experience from the observer’s point of view [12].

In this context, the narrative methodology holds significant potential in understanding the experiences of individuals living with pain [13]. This methodology allows researchers and clinicians to glean insights from individuals’ personal stories, allowing them to highlight crucial aspects of their perspectives by using their own words [14,15]. Examples of prior studies using narrative methodology included the study by Noel et al [16] who interviewed parents of young people with chronic pain, using a mixed pain sample to extract and analyze the patient’s narratives. Similarly, Meldrum et al [17] conducted a narrative analysis with semistructured interviews with children experiencing chronic pain, both at preintervention and 6-12 months post clinic intake. The use of narrative methodology has extended to written texts as well. McGowan et al [18] solicited written narratives (WNs) from women with chronic pelvic pain, while Dysvik et al [19] examined WNs from individuals with chronic pain 6 years after completing a pain management intervention. More recently, WNs were used to explore the experience of children with functional abdominal pain and their parents [20], adults with neck or back pain [21], and people with fibromyalgia [22]. In a related manner, Kathan et al [23] used a qualitative survey in which people were asked to respond to questions about pain acceptability by using their own words.

The growing interest in WNs is mainly due to the fact that asking for writing content to people suffering from a condition offers distinct advantages over oral inquiries. Primarily because writing facilitates the organization of ideas related to complex emotional experiences such as pain [24]. Additionally, WNs offer a time-efficient way to explore the subjective nature of pain, as individuals can complete the writing outside of health care consultations, making it an accessible initial approach to this complex phenomenon [20]. However, on the other hand, analyzing the content expressed by people with pain can be time-consuming for clinicians and researchers. For this reason, this study explores the feasibility of using artificial intelligence (AI) and in particular large language models (LLMs) to assess WNs of people with chronic pain.

AI has been used in the assessment of the pain field in some studies, as presented in the recent review by Abd-Elsayedin et al [25]. In this review, studies were found to address the following purposes: (1) diagnostic aid, (2) modeling pain progression, (3) predicting pain treatment response, and (4) improving treatment and pain maintenance. To achieve these, most of the studies used machine learning techniques. Although none of the reviewed studies used LLMs, there are few works available using them for pain research. Vaid et al [26], used locally running, privacy-preserving LLMs capable of following plain language instructions to extract characteristics of musculoskeletal pain (such as location and acuity) from a heterogeneous collection of unstructured clinical notes. The study used multiple patient notes, coded by 2 health care professionals, and found great precisions of the system in classifying pain location and acuity. In another study, Shrestha et al [27] tested the responses of GPT to clinical questions and recommendations based on an established clinical guideline. They found that the system was able to make clinical recommendations for low back pain, although it was not exempt from errors. In the same line, Gianola et al [28] tested the ability of GPT against clinical practice guidelines to answer clinical questions about lumbosacral radicular pain. They found negative results since the internal consistency was low, as well as the precision to follow the clinical guidelines for recommendations.

Using LLMs for the assessment of WNs provides several advantages, such as decreasing assessment time for clinicians —where LLMs serve as clinician assistants—and allowing language flexibility—meaning our methodology can be applied to various languages. This study can contribute to the open debate about the potential application of LLMs for health. While there is existing literature supporting benefits such as the capabilities for analyzing massive data, there are also studies showing disadvantages such as inaccuracies with the use of LLMs [29].

To evaluate LLMs’ capacity to assess pain narratives we used the narratives provided in Serrat et al [22]. In our investigation, GPT-4 was used to assign scores, as well as the scores’ explanation, for pain severity and disability as expressed in the narratives. Subsequently, we conducted a quantitative analysis by comparing these scores with expert ratings reported by Serrat et al [22], using statistical measures such as Pearson correlations, root-mean-squared error (RMSE), the weighted version of Gwet agreement coefficient (Gwet AC2) [30], and Krippendorff α [31]. Additionally, a qualitative analysis of the scores’ explanation was performed by consulting 2 experts specialized in chronic pain, who evaluated the accuracy of GPT's pain assessment and its potential use for future pain WNs assessments. Altogether, the primary contribution of this paper is its pioneering exploration of applying LLMs for WN assessment, validated through both automatic and human evaluation methods. To the best of our knowledge, this paper is the first attempt at using LLMs to assess pain WNs.

## Methods

### Procedure

This study uses participant data from a previous qualitative study [22], which explored the value of WNs for understanding the experience of people with fibromyalgia. Specifically, we used patient WNs, their assessments by 2 experts, and patient answers on standardized questionnaires to reanalyze the data using GPT-4 and compare its outcomes with the previous results. We have chosen GPT-4 as it is widely considered the state-of-the-art proprietary LLM model, which has been successfully applied in several medical contexts. See, for example, a recent study by Goh et al [32] on the use of GPT for diagnostic reasoning.

### Dataset

A total of 46 people completed the WNs task in Serrat et al [22]. The inclusion criteria for these participants were (1) fulfillment of the 2010/2011 American College of Rheumatology Fibromyalgia diagnostic criteria [33] and (2) age of 18 years or older. The exclusion criteria were having terminal illnesses or programmed interventions that might interrupt the study.

In this study, we selected data from 43 participants, that is, the ones written in Spanish (3 participants who did the task in a different language were excluded). They were requested to write about their pain experiences with the objective of capturing their personal viewpoints. A sheet was provided to participants informing them about that, and the following cues/points to compose the narrative were provided as follows.

- Describe your pain now and how you manage it.
- Describe your pain over time: how it began, if it has changed or stayed the same, and what has affected changes over time.
- Describe your feelings and how pain has made you feel (in the family, work, and social contexts) over time.
- Describe how pain has affected your daily life over time.
- Describe whether pain has affected the way you see your future and the things you would like to do.
- Describe the treatments you have followed and what effect(s) they have had.

Participants were explained that these cues were just tentative, and they could choose what to explain. They were given the option to complete the task in the language most comfortable and convenient for them and to write by hand (in this case WNs were transcribed for the analyses) or by using digital methods [22]. Participants were also asked to complete the following questionnaires.

- Revised Fibromyalgia Impact Questionnaire (FIQR): A 20-item questionnaire that measures functional impairment over the last 7 days. It has 3 dimensions: physical dysfunction (scores from 0 to 30), overall impact (scores from 0 to 20), and intensity of symptoms (scores from 0 to 50). The total sum of these scores ranges from 0 to 100, and higher scores indicate a greater impact. The Spanish version shows adequate internal consistency (Cronbach α=0.93) [34].
- Hospital Anxiety and Depression Scale (HADS): A commonly used questionnaire that evaluates the severity of anxiety and depression symptoms by 2 scales (each consisting of 7 items). Scores on each scale range from 0 to 21, with higher scores indicating greater severity of symptoms. The Spanish version has shown adequate internal consistency both for anxiety (Cronbach α=0.83) and depression (Cronbach α=0.87) subscales in individuals with fibromyalgia [35].
- Tampa Scale for Kinesiophobia: A scale composed of 11 items, to be answered on a 4-point Likert scale. The scale quantifies fear of movement, injury, or reinjury. Its total scores can range from 11 to 44, where higher scores indicate a greater fear of pain and movement. The Spanish version shows adequate internal consistency (Cronbach α=0.79) [36].

In Serrat et al [22], 2 independent reviewers (with expertise in pain, 1 psychologist and 1 physiotherapist) assessed the level of severity and disability expressed in the WNs on a scale from 0=indicating absence to 10=representing maximum levels. To ensure consistency in their evaluations, severity was defined as “the perceived magnitude of fibromyalgia concerning pain and overall suffering conveyed in each participant text.” Disability was defined as “the perceived extent to which fibromyalgia disrupts the usual activities and life of the writers.”

### Ethical Considerations

Our analysis uses anonymized data from the study by Serrat et al [22]. They received ethical approval for performing their study from the ethics committee of the Vall d’Hebron Hospital, Barcelona (reference: PR[AG]99/2022). Participants were asked first to complete the FIQR, HADS, and Tampa Scale for Kinesiophobia self-reported measurements and then to write a WN to describe their pain experience. Participants in the Serrat et al [22] study were asked for their informed consent, according to which the collected data could be used only for research purposes. No compensation was provided to the participants by Serrat et al [22] study nor for the human evaluation performed in this study.

### Experiments With GPT

We tasked GPT-4 to evaluate the WNs one by one. Specifically, we prompted GPT-4 to provide a score for pain severity and disability, as well as an explanation for the scores. Both scores were requested on a scale from 0 to 10 to compare with human assessments performed by Serrat et al [22]. Pain severity and disability are among the main outcome variables assessed in the pain field, and 11-point scales are, as recommended by experts [9,10], used frequently in the field.

To test the stability of these responses, we repeat the experiment with GPT-4 10 times. Both the scores of the 10 trials and the explanations (randomly chosen from one of the trials) were then used for the evaluation phase. The specific prompting strategy used is displayed in Textbox 1 and was performed with automated calls to the OpenAI API with model gpt-4-0125-preview.

The prompt includes the following instructions.

- Analyze patient narratives written in Spanish.
- Score each narrative on a scale from 0 to 10 for both severity and disability.
- Provide explanations for each score in English.
- Pay attention to factors that might reduce perceived severity or disability, such as coping mechanisms or support systems.

We performed experiments between April 22, 2024, and April 24, 2024 with 2 different values for GPT’s temperature parameter. This parameter allows controlling the randomness in the answers of the LLMs. We use 0=less randomness and 1=average randomness, the latter is the default value of GPT-4.

**GPT-4 prompts used for the experiment.**

As an expert psychologist specializing in evaluating pain in patients diagnosed with fibromyalgia, you are tasked with analyzing patient narratives about their pain and then scoring them on a scale from 0 (indicating no severity or disability) to 10 (indicating maximum severity and disability). These patients’ explanations about their pain and how they feel it are all written in Spanish. The level of severity is defined as the perceived intensity of pain and overall suffering. Disability is defined as the extent to which fibromyalgia hinders patients’ usual activities and quality of life and is to be rated based on your interpretations of the patients’ texts. Scores should accurately reflect the severity and disability levels described in patient narratives without inflation. A holistic evaluation capturing the complexity of experiences is crucial. Pay attention to phrases indicating coping mechanisms, resilience, or mitigating factors that may reduce perceived severity or disability. Consider contextual understanding, including coping strategies, support systems, and adaptive behaviors, which may mitigate perceived severity and disability. Your role involves receiving a patient’s narrative, enclosed within triple slashes, and analyzing it. You are expected to return your analysis in JSON format, with the following keys: “severity_score” providing the scores for severity ranging from 0 to 10, “disability_score” providing the scores for disability ranging from 0 to 10, “severity_explanation” providing an in English explanation for the severity score and “disability_explanation” providing an in English explanation for the disability score.

### Expert Evaluation

We asked two pain management and assessment experts to analyze the scores given by GPT-4 and the corresponding textual explanations by using Qualtrics. Although they form part of the list of authors of this paper, they only were aware of the general objective of the study and the instructions provided for their task. After doing their task, they were given access to all the details of the study and the manuscript. They performed the task between April 26, 2024, and May 3, 2024. Specifically, we randomly chose 1 trial out of the 10 performed with GPT-4 temperature 1. Then we asked the 2 experts, first to read the original narratives, the scores, and their explanations given by GPT-4 (Textbox 2) for pain severity and disability. Second to assess on a 7-point scale (from strongly disagree to strongly agree) to what extent the explanation: (1) could have been written by a psychologist expert in fibromyalgia, (2) adequately represents the scores for severity or disability, and (3) they would use the score and explanation provided by GPT-4 for patient assessment. An example of the evaluation task given to the experts can be seen in Multimedia Appendix 1. Each expert was blinded to the assessment made by the other expert.

**Example of GPT-4 (temperature 1) explanation for a pain severity score of 4.**

Originally, the patient experienced high levels of pain rated between 8 and 9, indicating a severe impact on their life due to fibromyalgia. However, through their own research and changes in lifestyle and treatment approaches, they have managed to reduce the frequency and intensity of their pain flare-ups to 1 or 2 days and the pain level to between 3 and 4. This significant improvement suggests a reduction in the severity of their condition, despite the initial diagnosis and challenges. Their proactive approach to managing their condition, including dietary changes and avoiding certain environmental factors, has effectively reduced the severity of their symptoms.

### Evaluation

We performed a 4-stage analysis. First, we used SD analysis to test the stability of assessments given in 10 trials by GPT-4 for pain severity and disability. Second, we compared GPT-4 scores (for pain disability and pain severity) with experts’ scores (from Serrat et al [22]) and a naive baseline predictor (which always predicts the average experts’ score) using three strategies: (1) interannotator agreement (IAA) to quantify the agreement between GPT-4 and the experts; (2) RMSE to measure the average squared differences among GPT-4, expert scores, and the naive baseline; and (3) mean absolute error (MAE) to determine if GPT-4 systematically overestimates or underestimates the expert scores (ie, if GPT-4 exhibits bias). IAA was assessed using 4 coefficients to ensure data reliability: percent agreement, weighted percent agreement (a weighted version of percent agreement that takes into account the ordinal nature of the data), Krippendorff α [31], and Gwet AC2 coefficient [30].

Third, we compute descriptive statistics (in IBM SPSS) for the experts’ evaluation of the GPT-4 assessments, as well as IAA (in this case, Krippendorff α was not reported because of the significant imbalance in scores, particularly toward the higher end). Fourth, we tested correlations between GPT-4 assessments, expert assessments, and standardized pain measurements [22]. Fifth, we used statistical tests, in particular, 2-tailed *t* tests (in SPSS) and *z* scores, (implemented in Python, Python Software Foundation) to test for significant differences between some of our results.

Regarding stage 2 strategy 1, we chose to use four agreement coefficients to ensure more robust results when reporting the IAA. While percentage agreement gives an indication of a raw agreement among annotators, weighted percentage agreement provides insight into the degree of difference between scores when annotators disagree. On the other hand, Krippendorff α accounts for the possibility that disagreement among annotators may occur by chance. Gwet AC2 further refines this by limiting the pool of items that would lead to an agreement by chance. As a result, Gwet AC2 reports more accurate agreement in the case of imbalance annotation, that is, those annotations where some scores are little or not used. Our experiments show an imbalance in ratings towards higher categories (6-10), making the use of Gwet AC2 coefficient more suitable due to possibility of the interanimation prevalence paradox [37]. The metrics used in strategy 1, as well as the metrics used in the other strategies of stage 2, have been calculated using Python. In particular, the *scikit-learn* package was used for error measurement, and the irrCAC library [38] to measure the coefficients of agreement. The weighted percent agreement, Krippendorff α, and Gwet AC2 coefficient were measured with ordinal weight. For more details on how the ordinal weights are computed, we refer to the book *Handbook of Inter-Rater Reliability: The Definitive Guide to Measuring the Extent of Agreement Among Raters, 4th Edition* [30].

## Results

### GPT-4 Assessment Stability

The mean and SD for pain severity and disability were assessed for 10 trials at 2 different GPT-4 temperatures (0 and 1). At temperature 0, the mean severity score was 8.13 (SD 1.09) and the mean disability score was 7.25 (SD 1.28). At temperature 1, the mean severity score was 8.08 (SD 1.02) and the mean disability score was 7.33 (SD 1.32). These results suggest stability in both pain severity and disability across the different 10 trials and temperatures.

**Figure 1:**
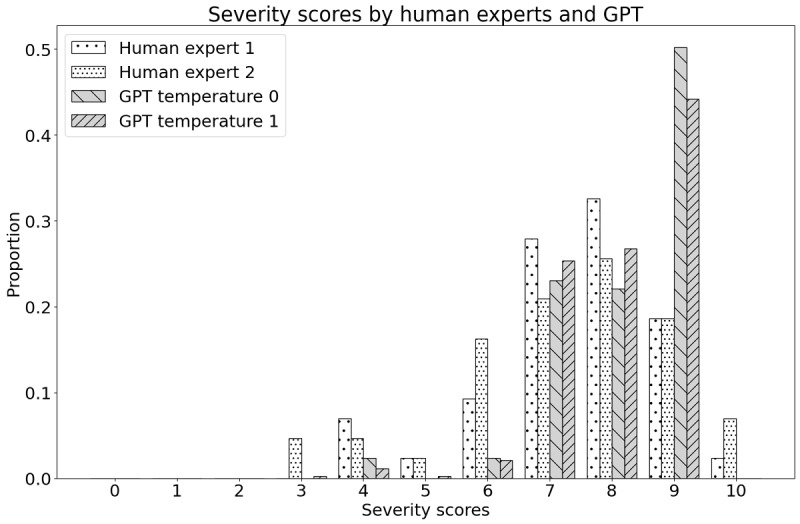
Distribution of the severity scores. Human experts versus GPT with 2 different temperature settings.

**Figure 2:**
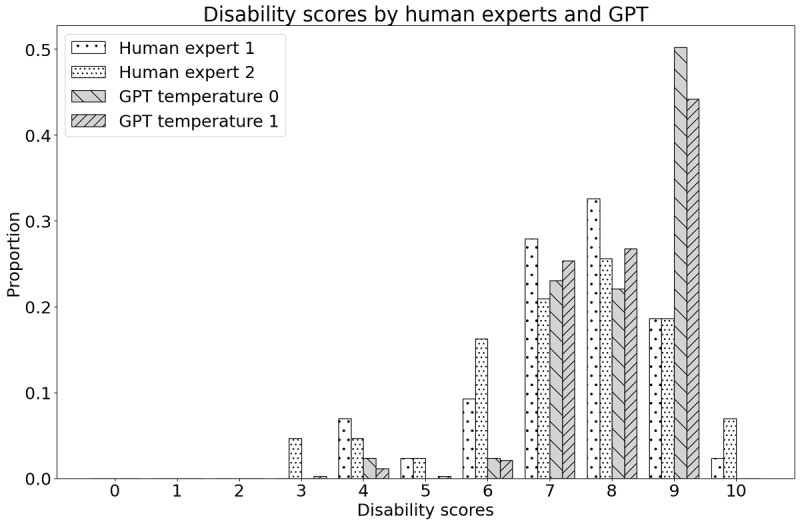
Distribution of the disability scores. Human experts versus GPT with 2 different temperature settings.

If we compare them with the average score of 2 human experts from Serrat et al [22], we observe that GPT has a slight tendency to overestimate the pain level and a lower SD. The mean for severity was 7.42 (SD 1.38) for expert 1 and 7.30 (SD 1.72) for expert 2, while the mean for disability was 7.00 (SD 2.12) for expert 1 and 6.95 (SD 2.05) for expert 2. This difference also becomes visible when comparing the distribution of the individual scores as depicted in Figure 1 for severity and Figure 2 for disability. GPT evaluations show a more expressed mode and are less frequent in scores lower or equal to 5. GPT also is reluctant to assign the highest score of 10.

### Agreement in Evaluations of Pain Severity and Disability Made by GPT and Experts

Results related to the IAA are presented in Table 1. IAA between experts is acceptable (both for pain severity and disability) considering Krippendorff α and Gwet AC2 coefficients. Delving deeper into the IAA analysis, the low percentage agreement (0.29 for pain severity and 0.31 for disability), combined with a high weighted percentage agreement (0.96 for pain severity and 0.95 for disability), suggests that while experts rarely chose exactly the same score, their disagreements were typically within adjacent scores. Given the subjectivity inherent in assessing WNs, which is influenced by numerous factors, the IAA demonstrates a satisfactory agreement among experts.

**Table 1:** Mean (SD) of the agreement between experts and GPT-4. Values for GPT-4 are averages (SDs) over 10 experiments.

			Expert 1 versus					Expert 2 versus	
			Expert 2		GPT-4 (temperature 0) (mean, SD)		GPT-4 (temperature 1) (mean, SD)	GPT-4 (temperature 0) (mean, SD)	GPT-4 (temperature 1) (mean, SD)
**Pain severity**									
	Percent agreement	0.29		0.36 (0.01)		0.36 (0.04)		0.27 (0.01)	0.32 (0.03)
	Weighted percent agreement	0.96		0.94 (<0.01)		0.94 (0.01)		0.95 (<0.01)	0.95 (<0.01)
	Gwet AC2	0.87		0.83 (<0.01)		0.84 (0.02)		0.84 (<0.01)	0.84 (0.01)
	Krippendorff α	0.66		0.46 (0.01)		0.45 (0.05)		0.51 (0.01)	0.49 (0.04)
**Disability**									
	Percent agreement	0.31		0.21 (0.02)		0.23 (0.04)		0.33 (0.02)	0.35 (0.08)
	Weighted percent agreement	0.95		0.94 (<0.01)		0.94 (0.0)		0.94 (<0.01)	0.94 (<0.01)
	Gwet AC2^a^	0.83		0.79 (<0.01)		0.79 (0.01)		0.8 (<0.01)	0.8 (0.02)
	Krippendorff α	0.69		0.47 (0.01)		0.49 (0.03)		0.57 (0.01)	0.57 (0.04)

^a^Gwet AC2: the weighted version of Gwet agreement coefficient.

When comparing GPT-4 scores with expert scores, Krippendorff α indicated slightly lower agreement compared to the agreement between the experts. However, percent agreement, weighted percent agreement, and Gwet AC2 are comparable to the agreement between experts, with the notable exception that, for pain severity (temperature 1), the percent agreement was higher than the one reached by the experts.

We compare the scores of pain severity and disability obtained with GPT-4 to those of the experts and the naive baseline using RMSE and MAE (by definition, MAE is 0 for the naive baseline).

GPT-4 can approximate the average of the expert ratings with RMSEs of around 1.20 for severity and 1.44 for disability (Table 2). These values are significantly lower than the ones obtained with the naive baselines with *P*<.001 for GPT-4 with temperature 0 and *P*<.01 for temperature 1. Furthermore, we can observe that the RSMEs between the 2 experts are very close to the ones obtained by GPT-4 when compared to the average of the 2 experts for disability and even smaller for pain severity. Overall, these errors are acceptable, especially when compared to the differences between the 2 experts.

**Table 2:** Predicting severity and disability scores with GPT with different temperature values. RMSE^a^ and MAE^b^ of GPT-4 compared to 2 expert evaluations and a naive baseline^c^.

	Statistical measures	Expert 1 versus					Expert 2 versus				Average of experts versus		
		Expert 2	GPT-4 (temperature 0)	GPT-4 (temperature 1)	Baseline	GPT-4 (temperature 0)		GPT-4 (temperature 1)	Baseline	GPT-4 (temperature 0)		GPT-4 (temperature 1	Baseline
**Pain severity**													
	RSME	1.15	1.27^d^	1.25^e^	1.38	1.39^d^		1.40^f^	1.72	1.20^d^		1.19^f^	1.45
	MAE	0.12	–0.71	–0.66	0.00	–0.83		–0.78	0.00	–0.77		–0.72	0.00
**Disability**													
	RSME	1.56	1.75^d^	1.74^f^	2.20	1.50^d^		1.53^f^	2.05	1.44^d^		1.44^f^	1.98
	MAE	0.05	–0.25	–0.33	0.00	–0.30		–0.37	0.00	–0.27		–0.35	0.00

^a^RMSE: root-mean-square error.

^b^MAE: mean average error.

^c^A significant difference between the root mean square errors of GPT-4 and the baseline (2-sided *P* value equivalent of the *z* score of 10 experiments with GPT-4 and the baseline).

^d^*P*<.001.

^e^*P*<.10.

^f^*P*<.01.

We also analyzed the MAEs (ie, expert score minus GPT-4 score), which indicate a potential tendency to underrate or overrate the expert scores. For both temperatures, we find that GPT-4 on average slightly overestimates the individual experts’ scores. More precisely, between 0.66 (temperature 1) and 0.83 (temperature 0) for severity and 0.25 (temperature 0) and 0.37 (temperature 1) for disability.

Finally, all the results commented on are quite similar when comparing temperatures 0 and 1. The comparison between GPT-4 scores and those provided by experts highlights a significant alignment in their assessments of WNs. This finding holds promise, especially given the inherent subjectivity involved in evaluating WNs. To delve deeper into this alignment, we enlisted 2 pain assessment experts to evaluate the GPT-4 scores and their accompanying descriptions.

### Expert Evaluation

The IAA among experts is acceptable, as shown in Table 3. The low percent agreement is compensated by a notably high weighted percent agreement (except for pain severity in question 1). This suggests that, although experts rarely assign the same score, they tend to choose adjacent ratings when a disagreement arises. This phenomenon indicating acceptable IAA is also reflected in Gwet AC2 values (except for disability in question 2).

**Table 3:** Agreement between experts on the 3 questions. Ordinal weights were then applied to these categories based on their positions on the scale [30]^a,b^.

		Question 1	Question 2	Question 3
**Pain severity**				
	Percent agreement	0.08	0.21	0.12
	Weighted percent agreement	0.50	0.95	0.91
	Gwet AC2	0.93	0.83	0.66
**Disability**				
	Percent agreement	0.17	0.21	0.33
	Weighted percent agreement	0.95	0.96	0.94
	Gwet AC2	0.81	0.43	0.72

^a^The coefficients were calculated by assigning numerical values to the categories: 1=strongly disagree, 2=disagree, 3=somewhat disagree, 4=neither agree nor disagree, 5=somewhat agree, 6=agree, and 7=strongly agree.

^b^This method acknowledges that experts may differ more significantly in their disagreement if one selects “somewhat disagree” while the other selects “agree” compared to if one chooses “agree” while the other chooses “strongly agree.”

As shown in Table 4, a 2-tailed *t* test analysis indicates that expert 2’s assessments were significantly higher (*P*<.001) for all 3 questions compared to those of expert 1’s. This phenomenon implies a divergence in experts’ scoring interpretations. Despite this difference, both experts appear to adhere consistently to their respective scoring criteria during the annotation process. This finding suggests that while there may be slightly individual variations in scoring approaches between experts, they maintain internal consistency in their assessments.

**Table 4:** Expert evaluation scores: mean (SD), max score is 7. Significant differences between the 2 experts are determined by a 2-sided *t* test^a^.

		Expert 1, mean (SD)	Expert 2, mean (SD)	*t* test (*df*)
**Question 1^b^**				
	Pain severity	5.72 (0.45)	6.88 (0.32)	–13.67 (84)^c^
	Disability	5.93 (0.26)	6.83 (0.37)	–13.10 (84)^c^
**Question 2^d^**				
	Pain severity	5.93 (0.26)	6.79 (0.41)	–11.62 (84)^c^
	Disability	6 (0)	6.79 (0.41)	–12.60 (84)^c^
**Question 3^e^**				
	Pain severity	5.44 (0.63)	6.72 (0.45)	–10.82 (84)^c^
	Disability	5.77 (0.43)	6.65 (0.48)	–8.99 (84)^c^

^a^The coefficients were calculated by assigning numerical values to the categories: 1=strongly disagree, 2=disagree, 3=somewhat disagree, 4=neither agree nor disagree, 5=somewhat agree, 6=agree, and 7=strongly agree.

^b^Question 1: The explanation could have been written by a psychologist expert in fibromyalgia.

^c^*P*<.001.

^d^Question 2: The explanation adequately represents the pain severity expressed in the narrative.

^e^Question 3: I would use the pain severity score and explanation above to help myself assess the patient's pain.

The mean assessment scores for both pain severity and disability are presented in Table 4. Assessments for the 3 questions were high (scores ranging from 5=somewhat agree and 6=agree for expert 1 and 6=agree and 7=strongly agree for expert 2) and with low variability (SD, ranging from 0 to 0.63). In other words, the experts agreed to consider GPT-4 assessments accurate and usable as clinician assistants.

In conclusion, the disagreement observed between the experts appears to stem from differing interpretations of scoring criteria, as evidenced by the consistent trend of 1 expert assigning higher scores than the other expert during disagreements, displaying a more optimistic outlook. This indicates a personal bias in their evaluation approaches. The identification of such a clear pattern in the evaluation process is particularly intriguing as it suggests a consistent trend in how assessments were conducted and interpreted by the experts. On the one hand, further analysis of this pattern could provide valuable insights into the underlying factors influencing the evaluation outcomes and shed light on the reliability and consistency of the assessment process. Understanding and leveraging such patterns can enhance the effectiveness and accuracy of automated systems like LLMs in pain narrative assessments. On the other hand, understanding this pattern in expert evaluations can provide valuable insights into the subjective nature of pain assessments and the potential impact on IAA in pain narrative evaluations.

Despite this pattern observed in the evaluation, both experts concur on the use of GPT-4 as a valuable tool for pain assessment tasks. This alignment in their assessment of GPT-4's effectiveness underscores its potential to complement and enhance traditional expert evaluations in pain narrative analysis.

### Correlations of Human and GPT-4 Assessments With Standardized Measurements

The mean of the ratings assigned by experts for severity and disability [22] significantly correlated with scores from the FIQR questionnaire, and the anxiety and depression scores from the HADS questionnaire. When analyzing the corresponding correlations with the scores given by GPT-4, for temperature 0, results were very similar to the ones found for experts with the exception that in this case there were no significant correlations between pain severity and disability and anxiety scores. However, the correlations between scores assigned by GPT-4 with temperature 1 and depression and anxiety, were in the same line as the ones found for the experts (Table 4). Also, in this case, the results indicate that GPT-4’s performance in assessing pain WNs closely approximates humans’ assessment. In addition, a further encouraging outcome is found: GPT-4’s pain WNs assessment shows a favorable alignment with standard pain assessment tests (for example, see GPT-4 with temperature 1 in the assessments of FIQR and HADS depression in Table 5).

**Table 5:** Pearson correlations of expert and GPT-4 assessments with standardized measurements^a^.

		FIQR^b^	TSK^c^	HADS^d^	
				Anxiety	Depression
**Experts**					
	Pain severity	0.41^e^	0.18	0.34^f^	0.44^e^
	Disability	0.44^e^	0.19	0.38^f^	0.46^e^
**GPT-4 (temperature 0) Mean of 10 Trials**					
	Pain severity	0.36^f^	0.17	0.25	0.35^f^
	Disability	0.42^e^	0.21	0.28	0.36^f^
**GPT-4 (temperature 1) Mean of 10 Trials **					
	Pain severity	0.43^e^	0.18	0.32^f^	0.41^e^
	Disability	0.49^e^	0.16	0.34^f^	0.45^e^

^a^Significant correlations are indicated with ^e^*P*<.01 and ^f^*P*<.05.

^b^FIQ-R: Fibromyalgia Impact Questionnaire.

^c^TSK: Tampa Scale of Kinesiophobia.

^d^HADS: Hospital Anxiety and Depression Inventory Scale.

^e^*P*<.01.

^f^*P*<.05.

## Discussion

### Principal Findings

Our preliminary study highlights the potential of using LLMs, such as GPT-4, for automatizing the assessment of pain severity and disability levels in patient WNs. WNs can be very useful for people’s pain assessment but are time-consuming for clinicians. The methodology based on LLMs presented in this paper conducts an automated assessment of the levels of pain severity and disability in the patient’s WNs. Our results indicate that experts in pain assessment can make use of LLMs for faster patient assessment. Indeed, the conducted analysis, bolstered by various statistical measures, reveals a significant resemblance between expert scores and those generated by GPT-4. This observation is further supported by the comparable correlation values observed between standardized measurements and assessments by both experts and GPT-4. Moreover, the positive reception from experts regarding the scores and explanations generated by GPT-4 underscores the potential applicability of automated systems in pain assessment. It is worth noting that both experts agree on the perceptions about GPT-4's scores and explanations: although one of them seems more positive in her assessments, leading to some variations in agreement indices.

### Limitations and Future Research

While these findings are promising, some limitations are present that motivate further research to advance this area.

First, in this study, we used pain severity and disability as main indicators of the texts, since we wanted to explore variables relevant in clinical context. International guidelines support measuring these in the pain field [9,10]. Moving forward, it would be beneficial to explore additional variables beyond pain severity and disability that could enhance the clinical relevance of automated assessments. For example, future research could instruct LLMs to assess levels of catastrophizing thoughts in the texts. These are common patterns of thinking in people with pain, related to a worse adaptation to pain in multiple studies, for example, Quartana et al [39]. Assessing this variable adequately would be very useful in the clinical context to identify people at risk of suffering more complex problems that need more attention.

Second, our findings suggest the need for ongoing efforts to enhance the precision of GPT-4 scores in pain WN assessments (with special attention to the extreme scores). While the agreement between GPT-4-generated scores and expert ratings was generally favorable, there is room for improvement. We observe that GPT-4 has a slight tendency to overestimate the pain severity and disability of WNs, using less frequent scores lower or equal to 5 than humans. Moreover, GPT-4 avoids using the highest score of 10. More research is needed to investigate if this pattern is observed with a greater sample of WNs or if it is linked to the sample used in this paper. If these persist in larger studies, a fine-tuning strategy can be considered to alleviate this problem. For example, future research should analyze the use of few-shot learning methods to test the performance of the GPT-4 or other LLMs in assessing pain WNs. By refining the training data and algorithms used by LLMs, we can strive to achieve even greater accuracy and reliability in automated pain WN assessments.

Third, this study, due to its preliminary nature, focused on people with fibromyalgia. Future research would benefit from including people with different chronic pain problems, and bigger samples to compare the performance of GPT-4 in different pain conditions. Big differences are not expected, since we assume that the ability of GPT-4 to analyze WNs would not depend upon the specific pain problem. However, more empirical tests are needed to support this assumption and extend our methodology to other pain and health problems.

Fourth, the use of GPT has some inherent limitations. For example, it may generate text that suffers from biases, hallucinations, and inconsistency [40,41]. For this reason, any tools that use GPT, such as the one we propose in this paper, should not be used as stand-alone tools; they always need human supervision. In our case, this should be supervised by clinicians. Additionally, future work could use the Quality Analysis of Medical Artificial Intelligence tool [42] which provides a standardized way to evaluate medical AI output as we have been analyzing. Future research should adopt this methodology when assessing the explanations given by LLMs about the assessment of the WNs. Finally, as previously explained, we asked for an explanation of the scores given by GPT-4 but this was not the case for the scores given by the experts in Serrat et al [22]. In future research, it would be interesting to compare the explanations of scores provided by an LLM and the corresponding explanations provided by human experts. For example, a blind annotation can aim to evaluate if experts would be able to distinguish the origin of these explanations.

## Data Availability

Nontextual data (expert and GPT scores) are included in this published article and Multimedia Appendix 1. The other materials generated or analyzed during this study are available from the corresponding author on reasonable request.

## Appendix

Multimedia Appendix 1 Example of the evaluation task given to the experts.

## Abbreviations

AI: artificial intelligence
FIQR: Revised Fibromyalgia Impact Questionnaire
Gwet AC2: the weighted version of Gwet agreement coefficient
HADS: Hospital Anxiety and Depression Scale
IAA: interannotator agreement
LLM: large language model
MAE: mean absolute error
RMSE: root-mean-squared error
WN: written narrative
