# Supplementary material for: Exploring the Capacity of Large Language Models to Assess the Chronic Pain Experience: Algorithm Development and Validation
Source: J Med Internet Res. 2025 Mar 31;27:e65903. doi: 10.2196/65903 (PMC11997525; doi:10.2196/65903)
Supplement: Multimedia Appendix 1 [file jmir_v27i1e65903_app1.pdf]

introduction

Thank you for taking part in this study.

For this task, you will be presented with a narrative from a fibromyalgia patient. You will then be asked to answer eight questions about it.

An Artificial Intelligence model scored the narrative for pain severity and disability on a scale of 0 to 10, explaining its scores choice. The model was instructed to perform the task given the following definition of pain severity and disability:

The level of severity, defined as the perceived intensity of pain and overall suffering, and disability, defined as the extent to which fibromyalgia hinders patients' usual activities and quality of life, are to be rated based on your interpretations of the patients' texts.

Your task is to evaluate the model explanations based on the narrative and scores. For each variable (that is, pain severity and disability), you will be asked to rate the explanation quality and usability on a seven-point scale. Lastly, you will be asked to provide (if needed) suggestions for improving the explanations.

In total you will be provided with 43 narratives.

ID_2

Narrative

For privacy reason, we can not publish the narrative

Pain Severity score: 8
Explanation for the pain severity score: The patient describes a long history of pain and various associated health issues since 2004, including damage to their cervical spine, both carpal bones, reflex sympathetic dystrophy of the right foot with permanent consequences, a recent right hand prosthesis, osteopenia, and irritable bowel syndrome. Additionally, the patient reports experiencing anxiety, depression, asthenia, insomnia, generalized joint, muscle, and nerve pain, muscle weakness, and severe fatigue. These symptoms indicate a high level of pain severity. The ongoing use of tramadol for pain relief and the history of other medications suggest significant attempts to manage this pain. The lengthy period of enduring these

health problems and the comprehensive impact on the patient's life highlight the severity of their condition.

	Strongly disagree	Disagree	Somewhat disagree	Neither agree nor disagree	Somewhat agree	Agree	Strongly agree
The explanation could have been written by a psychologist expert in fibromyalgia.	○	○	○	○	○	○	○
The explanation adequately represents the pain severity expressed in the narrative.	○	○	○	○	○	○	○
I would use the pain severity score and explanation above to help myself assess the patient's pain.	○	○	○	○	○	○	○

Disability score: 7

Explanation for the disability score: The patient is still actively working, engaging in rehabilitation exercises, and

practicing yoga, walking, and meditation, which reflects a considerable effort to maintain functionality despite their condition. However, they mention struggles with work due to physical demands exacerbating their condition, leading to increased anxiety and concerns about future abilities to cope, especially given their financial responsibilities. The support from their current partner and their determination to find a more suitable job suggest they are still managing to maintain some level of daily activity. However, the mention of significant memory worries and a family history of Alzheimer's disease adds to their burden. Their narrative demonstrates a determined coping mechanism but also suggests a substantial level of disability in managing everyday tasks and responsibilities without experiencing significant distress or hindrance.

	Strongly disagree	Disagree	Somewhat disagree	Neither agree nor disagree	Somewhat agree	Agree	Strongly agree
The explanation could have been written by a psychologist expert in fibromyalgia.	○	○	○	○	○	○	○

	Strongly disagree	Disagree	Somewhat disagree	Neither agree nor disagree	Somewhat agree	Agree	Strongly agree
The explanation adequately represents the disability expressed in the narrative.	○	○	○	○	○	○	○
I would use the disability score and explanation above to help myself assess the patient's pain.	○	○	○	○	○	○	○

Powered by Qualtrics
